# Small Bowel Obstruction Possibly Related to Migration of Trichobezoar Fragments Following Endoscopic Treatment

**DOI:** 10.1111/ped.70458

**Published:** 2026-06-15

**Authors:** Kana Maejima, Emiri Kaji, Satomi Nishimoto, Noriaki Sugawara, Taro Iwatsubo, Hideki Tomiyama

**Affiliations:** ^1^ Department of Pediatrics Osaka Medical and Pharmaceutical University Hospital Osaka Japan; ^2^ Endoscopy Center Osaka Medical and Pharmaceutical University Hospital Osaka Japan; ^3^ 2nd Department of Internal Medicine Osaka Medical and Pharmaceutical University Osaka Japan; ^4^ Department of General and Gastroenterological Surgery Osaka Medical and Pharmaceutical University Hospital Osaka Japan

**Keywords:** endoscopic treatment, small bowel obstruction, trichobezoar

A trichobezoar is an indigestible mass comprising ingested hair that forms in the stomach. Cases among adolescent females have been increasingly reported [1, 2]. Trichobezoars may migrate distally, extending from the stomach into the small intestine and causing small bowel obstruction. Although rare, this complication is clinically important [2]. Surgical removal has been the standard treatment for trichobezoars; however, advances in endoscopic techniques have improved endoscopic removal in selected cases [3, 4]. Endoscopic fragmentation, however, carries a risk of distal migration of fragments. We report a case of an adolescent female with trichotillomania in whom small bowel obstruction developed possibly due to migration of trichobezoar fragments following endoscopic treatment, ultimately requiring surgery.

A 13‐year‐old girl presented with an abrupt onset of abdominal pain and nausea. On examination, a firm mass was palpable in the periumbilical area. Irregular hair growth without alopecia was noted on the right scalp.

Trichotillomania had been diagnosed 5 years prior. Although her symptoms had improved after psychiatric counseling, she discontinued follow‐up 3 years prior. One year before presentation, she experienced similar but self‐limited abdominal symptoms.

Laboratory tests revealed mild anemia and hypoalbuminemia. Abdominal radiography showed no air–fluid levels or free air. Contrast‐enhanced computed tomography (CT) revealed an approximately 29‐cm × 15‐cm heterogeneous intragastric mass with air and high‐density components (Figure 1a), consistent with a trichobezoar. Upper gastrointestinal endoscopy confirmed a large hair mass occupying the gastric cavity (Figure 1b) and extending beyond the pylorus into the duodenum.

**FIGURE 1:**
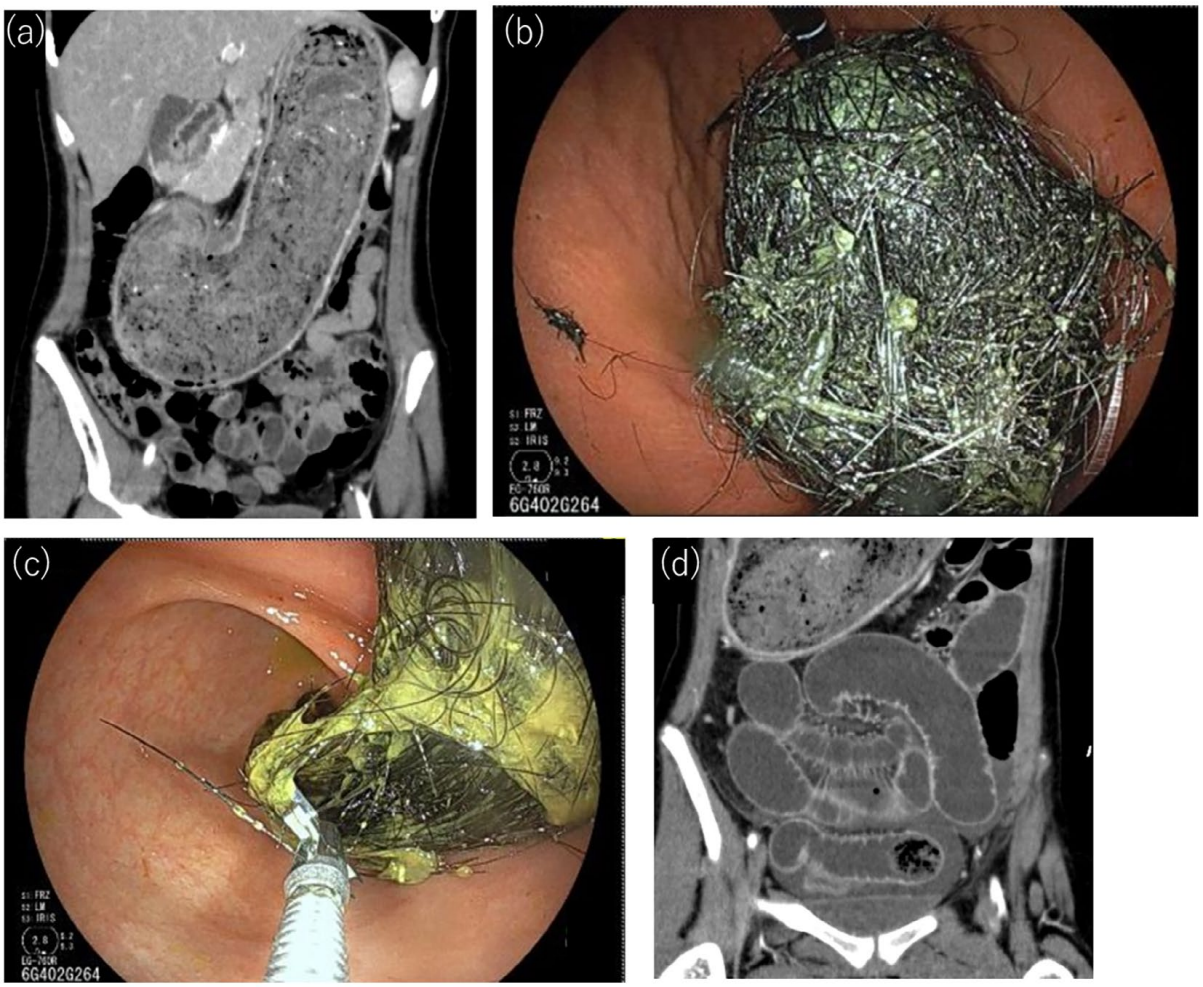
(a) Contrast‐enhanced abdominal CT showing a large heterogeneous intragastric mass containing air and high‐density components, consistent with a trichobezoar. (b) Upper gastrointestinal endoscopy demonstrating a large hair mass occupying the gastric cavity and extending beyond the pylorus. (c) Endoscopic fragmentation of the trichobezoar using a bipolar snare and electrocautery. (d) Repeat contrast‐enhanced CT showing small bowel obstruction due to migration of trichobezoar fragments to the ileum.

Endoscopic therapy was attempted at the patient's request. She was admitted for conservative management that included fasting and dissolution therapy comprising Coca‐Cola (The Coca‐Cola Company; Atlanta, GA, USA).

Endoscopic removal was attempted in five sessions over 10 days. Despite repeated attempts to cut the densely entangled hair using endoscopic scissors, a bipolar snare, and a bipolar electrosurgical knife with cautery, fragmentation was difficult because of the extremely compact structure of the bezoar (Figure 1c). Partial disintegration was achieved with bipolar electrocautery, and direct injection of Coca‐Cola into the bezoar was performed during the third session. However, only a small amount of material was retrieved during each procedure.

Complete endoscopic removal was deemed unfeasible; therefore, the patient agreed to surgery. Since the patient was able to eat solid food and had no vomiting or abdominal pain, it was determined that elective surgery was possible, and the patient was temporarily discharged while awaiting the procedure, with no additional abdominal imaging performed prior to discharge as there were no clinical signs suggestive of distal obstruction. Nine days after discharge, she presented with recurrent abdominal pain and vomiting. Repeat contrast‐enhanced CT demonstrated small bowel obstruction caused by trichobezoar fragment migration to the ileum (Figure 1d). The obstruction initially improved with bowel rest and intravenous fluids. Subsequently, laparotomy was semi‐emergently performed, and a residual 580‐g trichobezoar was removed through a small umbilical incision after dividing it into sections. Operative time was 3 h and 29 min. Her postoperative course was uneventful. Psychiatric follow‐up was resumed to address trichotillomania and prevent recurrence.

Small bowel obstruction is an uncommon complication of trichobezoar, occurring when a hair mass extends beyond the pylorus or migrates distally into the small intestine. Although endoscopic therapy has gained attention as a minimally invasive alternative to surgery, trichobezoar fragmentation is associated with the risk of distal migration and subsequent intestinal obstruction. Small bowel obstruction during endoscopic treatment of phytobezoars has been reported [4]; however, such complications during endoscopic treatment of trichobezoars are extremely rare. This case suggests that endoscopic fragmentation of a trichobezoar may result in distal migration of fragments and subsequent small bowel obstruction. Careful intraprocedural assessments and close postprocedural monitoring are essential when performing endoscopic therapy.

## Author Contributions

Kana Maejima and Emiri Kaji drafted the initial manuscript. Kana Maejima, Emiri Kaji, and Satomi Nishimoto were responsible for the overall clinical management of the patient. Noriaki Sugawara and Taro Iwatsubo performed the endoscopic treatment and contributed to the acquisition of imaging findings. Hideki Tomiyama performed the surgery. The final manuscript was reviewed and approved by all authors.

## Consent

Oral informed consent for publication of this case report and the accompanying images was obtained from the patient and her parents.

## Conflicts of Interest

The authors declare no conflicts of interest.

## Data Availability

The data that support the findings of this study are available on request from the corresponding author. The data are not publicly available due to privacy or ethical restrictions.
